# The path to precision medicine for MS, from AI to patient recruitment: an interview with Mauricio Farez and Helen Onuorah

**DOI:** 10.1038/s42003-021-02402-9

**Published:** 2021-07-22

## Abstract

This year’s World Brain Day is focused on stopping Multiple Sclerosis (MS). Although amazing progress has resulted in the development of relatively successful MS therapies, access to such therapies is a major problem for most of the world. In addition, major advances are still needed that would enable more precise treatment of MS for all patient demographics. We therefore spoke to Dr Maurico Farez, whose pioneering work focuses on the use of AI for precision medicine in MS and Helen Onourah, who has highlighted crucial issues surrounding the inequities that exist in MS research.

**Figure:**
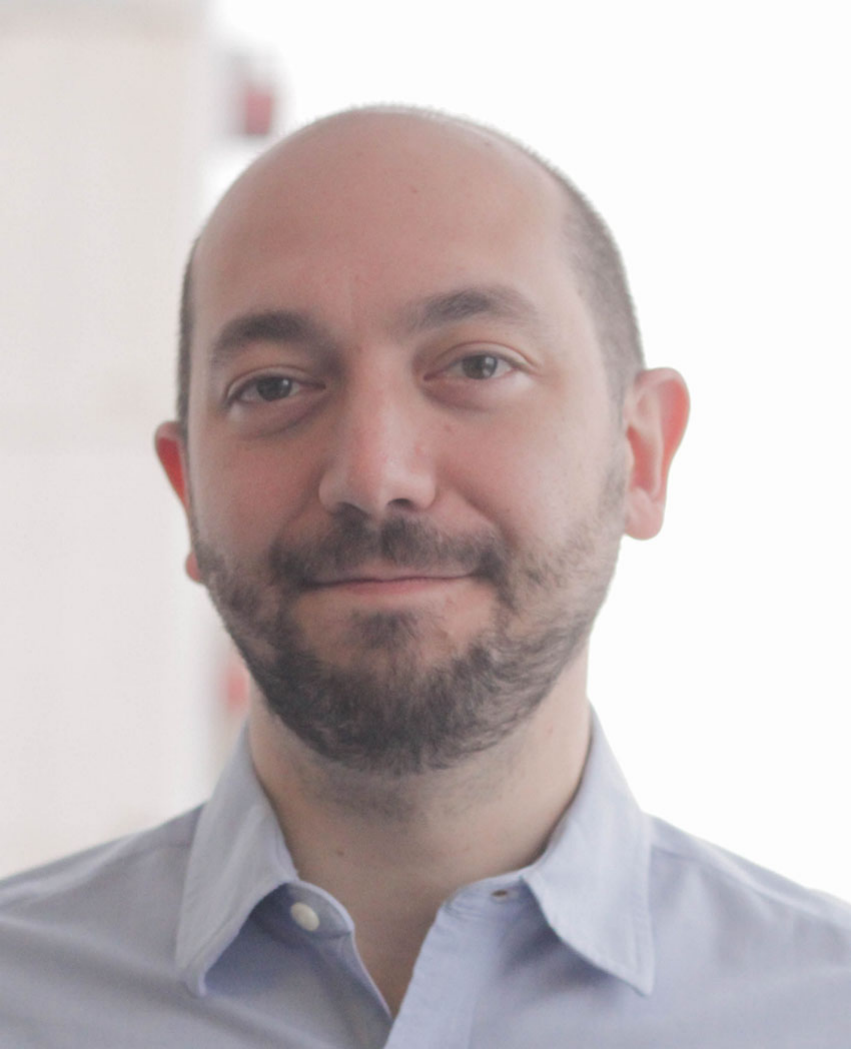
Maurcio Farez

Mauricio Farez is the Co-Founder of Entelai, a company providing AI services for the healthcare industry and also currently holds a position as an Independent Researcher of CONICET-FLENI. Mauricio received his M.D. degree with honors in 2006 and continued his training as a Research Fellow in Dr. Howard Weiner’s laboratory at Harvard Medical School followed by a Master of Public Health at Harvard School of Public Health. He has received several awards including the Bruce S. Schoenberg International Award in Neuroepidemiology, the Young Investigation award of the Argentinean Multiple Sclerosis Society and the Du Pre award from the Multiple Sclerosis International Federation. He is coauthor of 68 papers in international peer reviewed journals and has contributed to several book chapters. He is currently focused on solving modern healthcare challenges through the use of artificial intelligence.

**Figure:**
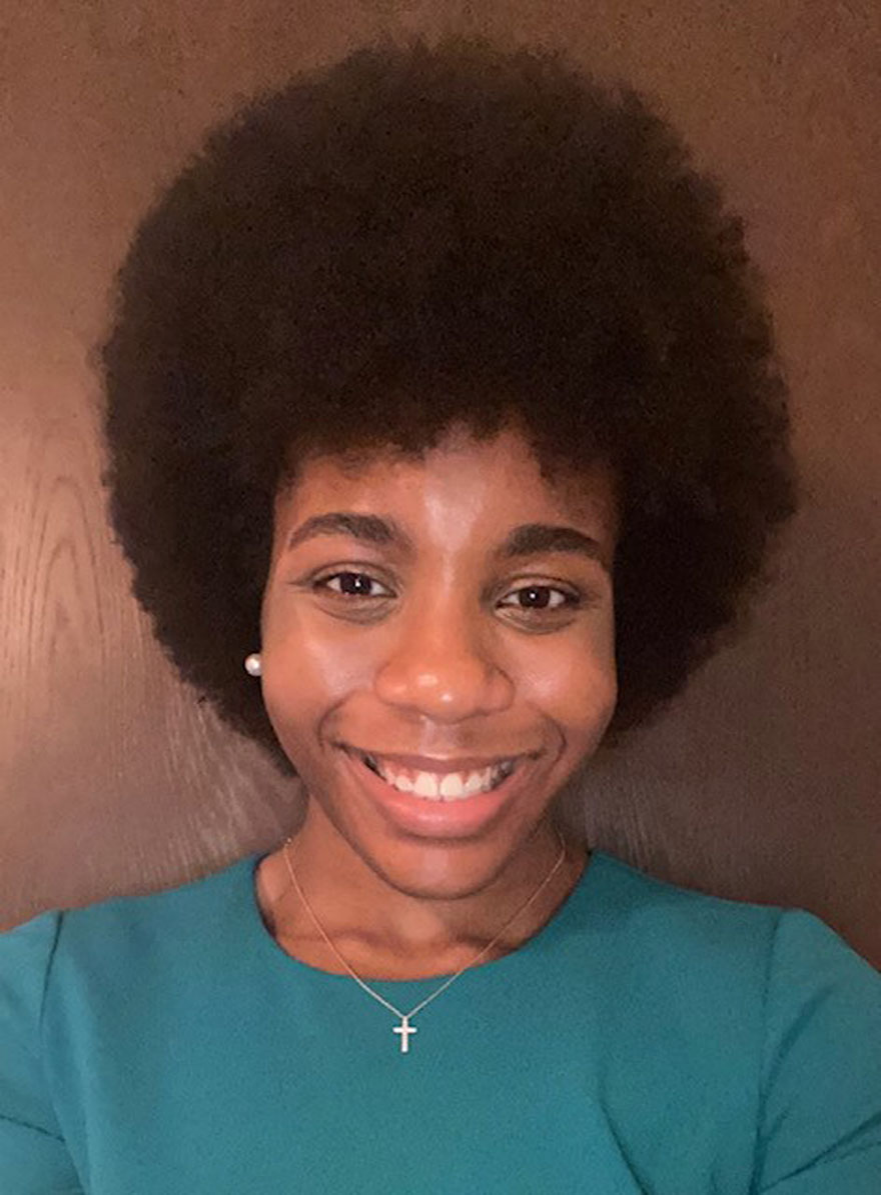
Helen Onuorah

Helen Onuorah is a research assistant at the University of Texas at Austin’s Dell Medical School working in the Multiple Sclerosis Imaging and Outcomes Lab headed by her ‘superhero’ Dr. Leorah Freeman. She received a BA in Neuroscience with a minor in Chemistry from the University of Pennsylvania in 2020, and is currently applying to medical school. In her spare time she enjoys drawing and playing the piano, and also spending time with her family.

Dr Farez, please tell us about your research

[MF] In the early stages of my career I was trained and worked mainly in basic MS research involving different biological pathways of relevance in T cell biology and disease activity. I did this both in animal models and patients. As I obtained my training in public health, I started moving from population evidence or findings, to the molecular levels: I love to take a clinical or radiological observation in MS patients and figure a biological explanation all the way to the gene expression level. More recently, I have been using machine learning and artificial intelligence to make those clinical and radiological observations more quantitative and ready to use in translational research.

What achievements have you been most proud of?

[MF] A researcher’s life is full of small victories or achievements (also big disappointments when experiments don’t work!). One achievement I am proud of is that by using the approach of relevant population-level observation, we were able to find an interesting pathway for melatonin-related genes in MS patients. Specifically we showed a seasonal fluctuation in relapses to the exact genetic level. Another great achievement, I would say is that, as a MS researcher, but also as a physician taking care of patients, I was always frustrated with the lack of precision in measuring key parameters such as lesion number and volume and brain volume/atrophy in my patients. How could I say something about treatment efficacy if it was based on a qualitative assessment using a printed MRI scan of 3 × 3 cm? Using AI, we developed, validated, and implemented algorithms to perform such measures with Entelai, a company I co-founded. Now, we can precisely measure brain and lesion volumes in our patients with AI within minutes, and we are using this information not only to make better clinical decisions, but for several translational and collaborative research projects.

Having established yourself in the field of MS research, where do you see it going over the next decade?

[MF] I am happy to see that it is going to a more quantitative and precision medicine-based approach. Artificial intelligence is revolutionizing every field of medicine and it is slowly growing in the MS field and, without doubt, it will occupy the headlines in the next few years in the context of more precise and faster measurements, and helping us in differential diagnosis or to uncover new variants or diseases. Every lab will have a data scientist working with deep learning models and using this powerful tool to answer new questions (in particular for images and genetic data). I do hope that we will also work more on the cause of MS. Without knowing the exact cause we are not getting closer to a cure. We need to understand why people get MS and focus on this. So my wish for the next decade is that funding agencies and donors devote time and resources for this challenge.

One of the key issues faced when developing precision medicine is ensuring that patient recruitment covers all demographics affected by the disease. Helen, please tell us about your research at the moment

[HO] My research focuses on the inequities that exist in MS research and access to specialized MS care. For over a year, I have examined the racial inequalities in phase III trials of MS disease modifying therapies (DMTs) and found that race is underreported in phase III trial publications and that non-White people with MS are largely underrepresented in the participant pools of these trials. Recently, I began a project that seeks to improve the health literacy of the MS patients seen at our clinic by firstly capturing their unique backgrounds, as well as their perspectives on MS and concerns about their disease management. With this insight we hope to develop inclusive culturally-sensitive MS resources that enhance health literacy while allowing people with MS to be confident advocates for their health.

What inspired you to get involved in MS research at such a young age?

[HO] I was first introduced to MS research while working at Penn as a clinical research assistant. My work was mainly behind the scenes–that is, patient scheduling and preparing tubes for collection of biological samples. I enjoyed that work and picked up bits and pieces about what MS is, but I wanted to be more hands-on with the research. When I joined the team at UT, I learned that MS is an incredibly heterogeneous disease–you never know what you’re going to get when a patient walks in the room. I became interested in how this heterogeneity manifests in diverse populations, but was surprised to discover there wasn’t much research on this topic. This segwayed into my work on inequalities in MS research.

Helen, you have already made an impact at such an early stage of your career. What are your aims for the future?

[HO] I plan to attend medical school next year and become a neurologist. I hope that I can continue to do work that highlights the blind spots in MS research and patient care in order to promote inclusive research practices and equitable healthcare access.

*Interviews conducted by Associate Editor Karli Montague-Cardoso for World Brain Day 2021*

